# Reversible cataract after exposure to distilled water: a case report

**DOI:** 10.1186/s12886-018-0810-6

**Published:** 2018-07-20

**Authors:** Young Hoon Yang, Bu Ki Kim, Su Joung Mun, Hyun Tae Choi, Young Taek Chung

**Affiliations:** Onnuri Eye Clinic, 325, Baekje-daero, Wansan-gu, Jeonju-si, Jeollabuk-do South Korea

**Keywords:** Distilled water, Anterior subcapsular cataract, Reversible cataract, Full-thickness astigmatic keratotomy, SMILE

## Abstract

**Background:**

To report a case of a reversible cataract after intracameral infusion of distilled water during full-thickness astigmatic keratotomy.

**Case presentation:**

A 20-year-old male whose bilateral anterior chambers were exposed to distilled water during astigmatic keratotomy developed bilateral corneal edema, inflammation of the anterior chambers, and an anterior subcapsular cataract in the right eye. After 1 month of topical administration of 0.1% prednisolone acetate and 5% NaCl, the bilateral inflammation of the cornea and anterior chambers cleared; endothelial cell density decreased by 41.1% in the right eye and 12.7% in the left eye. The cataract in the right eye decreased centripetally. Small incision lenticule extraction surgery was performed at 2 months after the astigmatic keratotomy; the patient’s uncorrected distance visual acuity 3 months later was 20/25 in both eyes.

**Conclusions:**

This case suggests that a cataract that develops as a result of instantaneous intracameral exposure to distilled water is reversible.

## Background

Distilled water is widely used in operating theaters to clean surgical instruments. Because it is transparent and odorless, it may be difficult for surgical personnel to distinguish distilled water from normal saline or balanced salt solution (BSS). There is always a risk that distilled water will be loaded into a syringe, and there have been reports of corneal endothelial damage resulting from inadvertent infusion of distilled water during cataract surgery [1, 2]. As distilled water is very hypotonic, with 0 osmolality, and has no protective ion composition, buffering capacity, or antioxidant properties for intraocular tissues, unlike aqueous humor or BSS, intraocular infusion of distilled water may damage intraocular tissues, including the corneal endothelium.

Intraocular infusion of distilled water may also affect the lens of a phakic eye. There have been experimental reports of changes in intralenticular osmolality and hydration in various hypotonic media [3, 4]; however, to our knowledge, there has been no in vivo report of the effects of intraocular infusion of distilled water on the lens of a human phakic eye. Thus, we report a case of a reversible cataract after inadvertent intracameral infusion of distilled water during full-thickness astigmatic keratotomy.

## Case presentation

A 20-year-old male was admitted to our clinic for refractive surgery. The patient’s uncorrected distance visual acuity (UDVA) was 20/300 in both eyes, and the best corrected distance visual acuity (BCDVA) was 20/20 in both eyes (right eye − 3.50 –4.00 × 180, left eye − 3.00 –5.50 × 175). The patient’s cornea and lens were clear under a slit-lamp microscope. Specular microscopy showed no morphological abnormality of the corneal endothelial cells, with an endothelial cell density of 3145 mm^2^ in the right eye and 3165 mm^2^ in the left eye. With a diagnosis of complex myopic astigmatism, the patient was scheduled to have small incision lenticule extraction (SMILE) surgery after the astigmatism was reduced by full-thickness astigmatic keratotomy in both eyes.

Bilateral astigmatic keratotomy was performed on the right and left eyes with informed consent. Briefly, 5.7-mm-long full-thickness incisions were made in the 12:00 o’clock direction in the right eye and the 12:05 o’clock direction in the left eye; the rest of the surgery was performed as described previously [5]. During the surgery, anterior chamber reformations were performed by infusing 12 mL of syringe fluid into the right eye and 3 mL of syringe fluid into the left eye. Shortly afterwards, a decrease in corneal transparency and opaque anterior chambers in both eyes were noted under a surgical microscope. We examined the infused fluid and found that distilled water had mistakenly been loaded into the syringe instead of BSS. The anterior chambers of both eyes were promptly irrigated thoroughly with BSS, and the surgery was completed.

Immediately after surgery, very dense superficial punctate keratitis appeared in both eyes, involving the entire cornea. The anterior chamber reactions were difficult to observe, and moderate corneal edema was evident. The patient complained of blurred vision with mild pain in both eyes. Topical 1% prednisolone acetate (Pred Forte®; Allergan, Westport, Ireland) and 5% NaCl (Muro128®; Bausch and Lomb, Rochester, NY, USA) eye drops were applied every 4 h to treat the bilateral inflammation induced by intracameral infusion of distilled water.

**Fig. 1:**
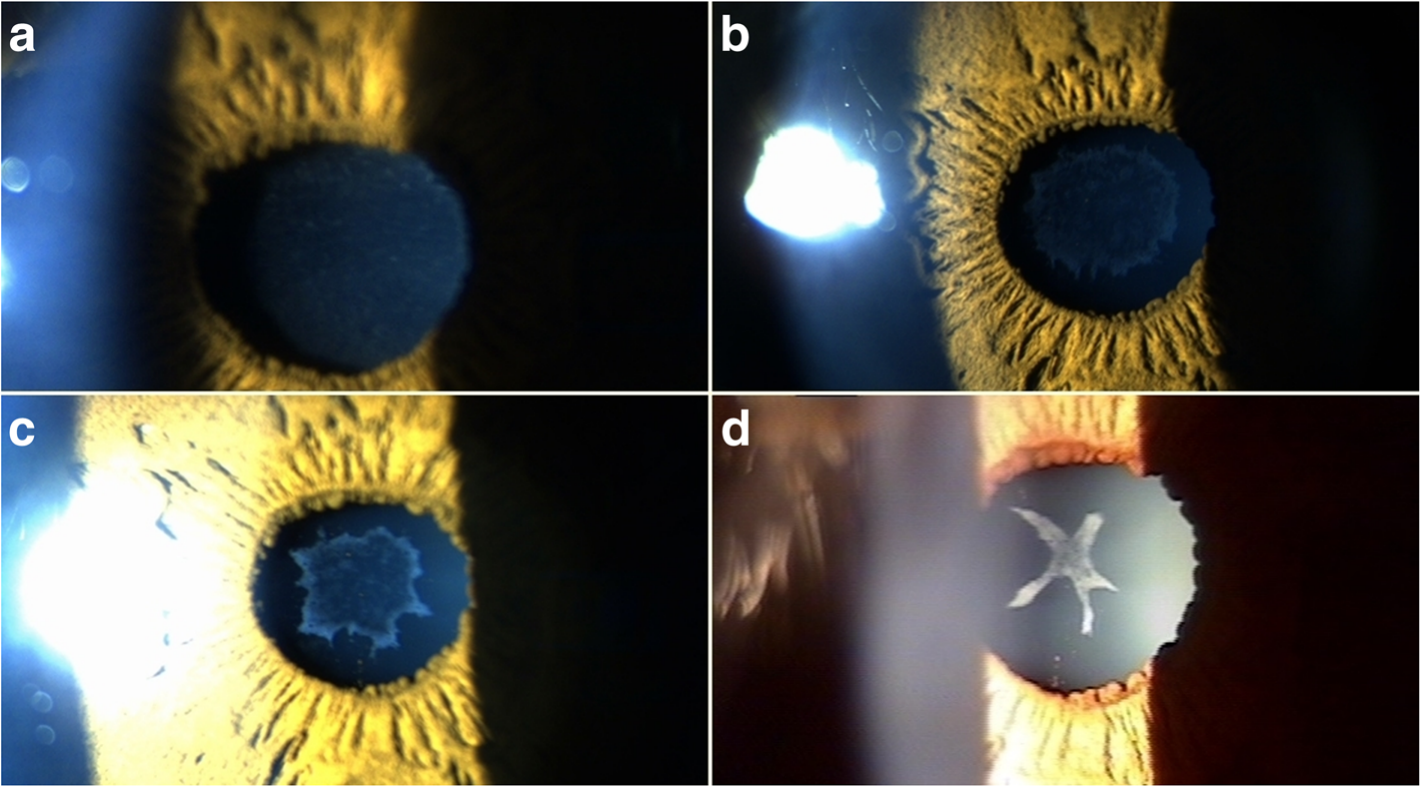
Photographs of an anterior subcapsular cataract in the lens of the right eye at 1 day (**a**), 1 week (**b**), 2 weeks (**c**), and 1 month (**d**) after astigmatic keratotomy. The cataract decreased in a centripetal pattern

**Fig. 2:**
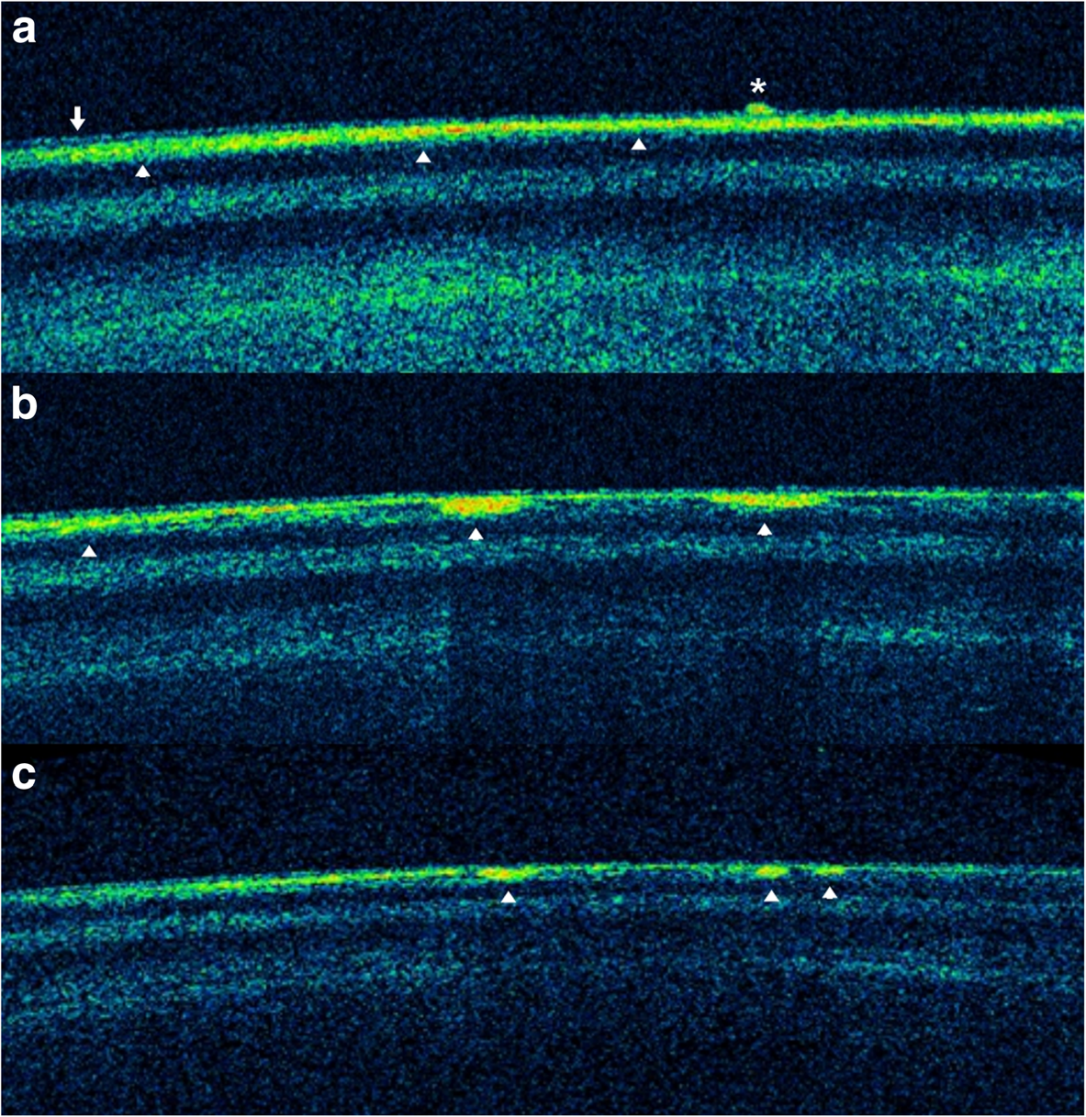
Anterior segment OCT images of the lens of the right eye at 1 day (**a**), 1 month (**b**), and 5 months (**c**) after astigmatic keratotomy. The anterior subcapsular cataract is seen with high signal intensity (arrowheads) below the anterior lens capsule (arrow). **a** Although the broad anterior subcapsular cataract is not clearly distinguishable from the anterior lens capsule, the lesion is under the capsule, in contrast with the inflammatory material on the capsule (asterisk). **b** The cataract is seen more clearly, although decreasing in a centripetal pattern. **c** The cataract decreased further, presenting with very small high-signal intensities

The next day, the patient’s BCDVA was 20/50 in the right eye and 20/40 in the left eye. Intraocular pressure was 12 mmHg in the right eye and 11 mmHg in the left eye. The patient still complained of blurred vision but had no pain in either eye. Corneal edema persisted with pachymetry showing a central corneal thickness (CCT) of 650 μm in the right eye and 580 μm in the left eye, but the extent of the diffuse superficial punctate keratitis decreased, with the anterior chambers showing reactions of 4+ in the right eye and 3+ in the left eye. Thin vacuolar anterior subcapsular opacity was seen in the lens of the right eye (Fig. 1a), although the lens of the left eye was clear. Anterior segment optical coherence tomography (OCT) confirmed the opacity was located beneath the anterior capsule (Fig. 2a). One week after surgery, the patient’s BCDVA was 20/30 in the right eye and 20/25 in the left eye. The corneal edema of both eyes decreased slightly with a CCT of 592 μm in the right eye and 555 μm in the left eye. The anterior chamber reactions in the right eye and left eye were 2+ and 1+, respectively. The thin anterior subcapsular cataract decreased in a centripetal pattern, allowing partial recognition of the clear lens in the nondilated state (Fig. 1b). Two weeks after surgery, the patient’s BCDVA was 20/25 in the right eye and 20/20 in the left eye. Mild corneal edema remained with a CCT of 560 μm and 523 μm, respectively, and the anterior chamber reactions in the right eye and left eye decreased to 1+ and trace, respectively. The cataract became smaller, showing a marginal moth-eaten appearance (Fig. 1c). One month after surgery, the corneal and anterior chambers were clear in both eyes. The cataract further decreased in size (Fig. 2b) and exhibited a starry appearance (Fig. 1d). The patient’s UDVA was 20/300, and the BCDVA was 20/20 in both eyes (right eye − 4.75 –1.25 × 10, left eye − 4.50 –1.00 × 175). Corneal endothelial cell densities were 1852 mm^2^ in the right eye and 2762 mm^2^ in the left eye.

**Fig. 3:**
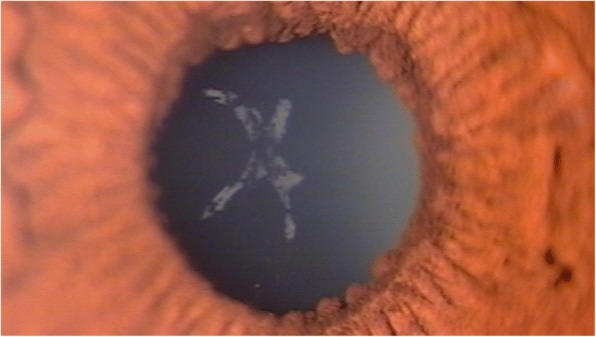
Photograph of an anterior subcapsular cataract in the lens of the right eye 3 months after small incision lenticule extraction surgery. The cataract was fainter than that in a photograph taken 1 month after the astigmatic keratotomy (Fig. 1d)

As the inflammation in the anterior segment subsided and refraction became stable in both eyes, SMILE surgery was performed at 2 months after the astigmatic keratotomy. One week after the SMILE surgery, the patient’s UDVA was 20/20 in both eyes. Three months after the SMILE surgery, the UDVA was 20/25 and the BCDVA was 20/30 in both eyes (right eye + 0.50–0.25 × 180, left eye + 0.25–0.25 × 180). No surgical complications, such as keratitis or keratectasia, manifested during the 3-month postoperative follow-up, and the subcapsular cataract of the right eye became fainter (Fig. 2c), clearly showing a decrease in the center and the 4 o’clock direction of the opacity (Fig. 3).

## Discussion and conclusions

The most interesting fact in this case is that a reversible cataract developed on exposure to distilled water. To our knowledge, this is the first case report of a reversible cataract after intracameral infusion of distilled water in a human phakic eye in vivo. Every case of intraocular infusion of distilled water reported to date has occurred during cataract surgery, so cases focused mainly on corneal endothelial damage and not the effects of the distilled water on the lens. One experiment examined volume regulation of the lens in various hyposmolar media [3], but did not set the concentration close enough to the 0 osmolality of distilled water; the study demonstrated change in the lens after long-term exposure, not instantaneous exposure as in our case.

The cataract in this case resembled glaukomflecken, a pathognomonic sign of a previous intraocular pressure spike in acute angle-closure glaucoma. Such increases in intraocular pressure may cause ischemia of the lens epithelial cells and degeneration of the subepithelial cortex, resulting in small, multiple, irregular, or patched, gray-dot opacities on the lens surface. However, the findings in our case were not relevant in relation to the increased intraocular pressure, which may have caused glaukomflecken in several circumstances. First, glaukomflecken opacities would not show reversible changes, although they are buried under newer, transparent layers of lens epithelial cells over time. Second, characteristic signs of acute angle-closure such as sectoral iris atrophy, decreased pupillary reactivity, and pigment dispersion on corneal endothelium were not found in our case. The patient also did not complain of intense eyeball pain, nausea, or vomiting during surgery. Third, while reforming the anterior chamber, we used cannula to evacuate the excessive irrigating solution by depressing the posterior portion of the keratotomy site. Thus, an acute and excessive increase in intraocular pressure was prevented. Last, we considered the possibility of ciliary block glaucoma by posterior misdirection of distilled water into the vitreous body, but the post-operative intraocular pressure was normal and the anterior chamber was well maintained without being shallow. There was also no sign of vitreous liquefaction that would have appeared as a result of distilled water contacting the vitreous body. We eliminated, therefore, the possibility of glaukomflecken in our case.

We also considered other factors such as a postoperative inflammatory reaction, cold distilled water, or fast aqueous flow changes for causing reversible cataracts, but concluded that none of these were likely to have a significant effect. We assumed that the postoperative inflammatory reaction led to a decrease in the BCDVA along with corneal edema, but the duration of the inflammation was not long enough to cause a cataract. We also eliminated the effect of cold distilled water because the infused distilled water was at room temperature. In the case of flow changes, it was unlikely that the flow would directly damage the lens, because the needle tip was not directed toward the lens while performing anterior chamber reformation. Furthermore, it was unlikely that the brief irrigation time would have caused the cataract by hindering the aqueous humor circulation and inducing a change in lens metabolism. We therefore considered distilled water exposure as the sole cause of the reversible cataract.

The effects on the lens from exposure to distilled water are caused by hyposmotic stress. Edema develops in the epithelial and fiber cells of the lens as a result of the influx of water secondary to the osmotic gradient, and low-index refraction fluid accumulates between the fibers of the lens. Intracellular edema changes the transparency of normally clear lens cells, causing potentially reversible opacity [6]. In an animal study on sugar cataract formation, Robison et al. reported that the epithelium is the area of the lens first affected by intracellular edema and reversible opacification [7]. If edema persists and damages the lens epithelium and fibers, it will alter the permeability of the cell membrane and disrupt active transport, accelerating the loss of lens clarity.

Cases of reversible cataract as a result of osmotic stress reported to date have mainly been associated with diabetes with an uncontrolled serum glucose level [8–11]. In these cases, intralenticular osmolality is abnormally higher than the surrounding aqueous humor and subjected to hyposmotic stress. When the glucose level of the aqueous humor increases following uncontrolled serum hyperglycemia, sorbitol accumulates inside the lens and intralenticular osmolality increases, leading to an influx of water and consequent swelling of the lens. When the osmotic gradient between the aqueous humor and lens expands with a sharp decrease in the serum glucose level following initiation of strict anti-hyperglycemic therapy, the hyposmotic stress on the lens worsens, accompanied by an additional influx of water. The lens swells further under this double osmotic shock and develops a cataract [8, 12]. However, in most cases the cataract is reversible if the serum glucose level stabilizes without fluctuation with the continuation of antidiabetic therapy.

In contrast, our case shows the development of a cataract in a lens with normal intralenticular osmolality encountering a single osmotic shock due to a sudden, abnormal drop in the osmolality of the surrounding aqueous humor. This shows that a sudden change in aqueous humor osmolality itself can result in a cataract, regardless of the value of the osmolality. In addition, the lower the osmolality at initial exposure, the harder it is to reverse the lens hydration and more severe the histological denaturation [3]. Moreover, our case showed development of a cataract in the right eye only, despite bilateral exposure to distilled water. It is hard to explain why the cataract developed in the right eye only, but we believe that more distilled water may have been infused into the right eye during the anterior chamber reformation and for a longer time, as the astigmatic keratotomy was performed from right eye to left eye.

**Fig. 4:**
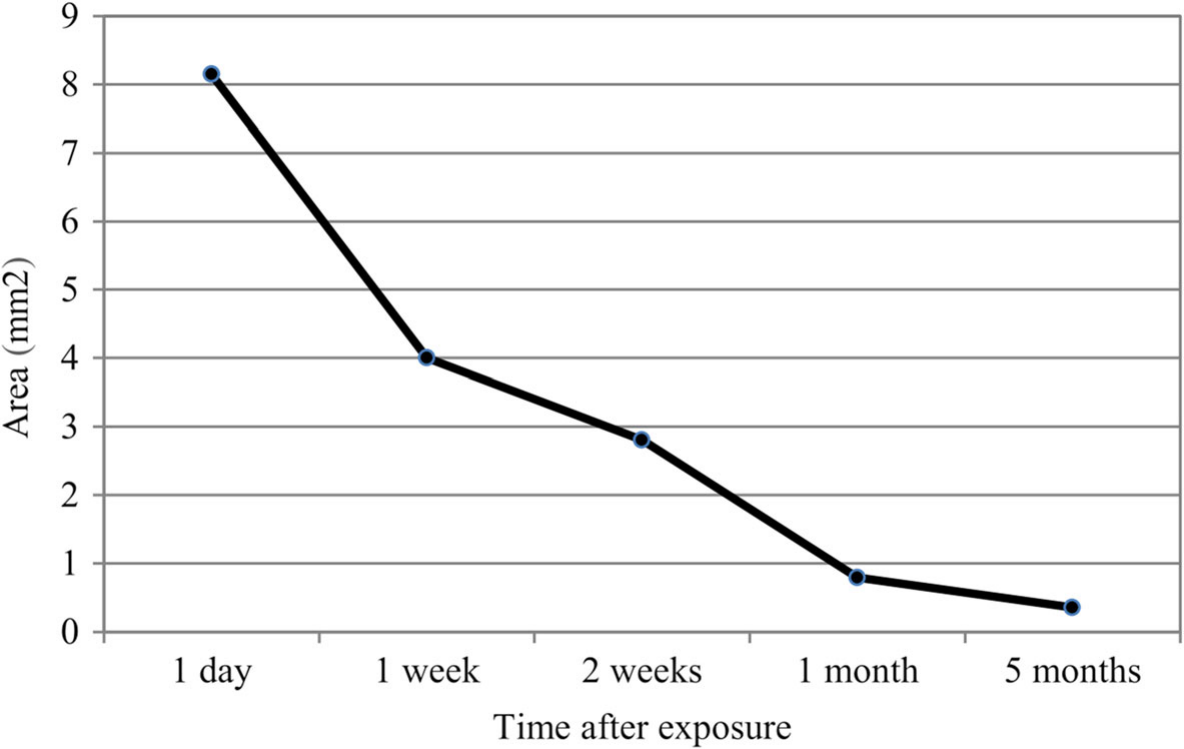
Changes in the surface area of the cataract after distilled water exposure in astigmatic keratotomy

The anterior subcapsular cataract in our case showed a thin and widespread fine vacuole pattern early on after exposure to the distilled water. The cataract decreased in a centripetal pattern as time passed, similar to cases of reversal of diabetic posterior subcapsular cataracts [8, 10]. We used anterior segment photography and anterior segment OCT to clarify the reversible change in the anterior subcapsular cataract. While taking anterior segment photographs, we used a single slit lamp setting to minimize bias by the slit beam direction and reflection. We used anterior segment OCT to eliminate inflammatory materials such as membranes or plaques that might overlap the cataract and significantly affect analyses of the photographs. We also quantified the reversible change by calculating the surface size of the cataract using ImageJ software (National Institutes of Health, Bethesda, USA). The surface sizes of the cataract, which decreased continuously at 1 day, 1 week, 2 weeks, 1 month, and 5 months after the astigmatic keratotomy, were 8.146 mm^2^, 4.001 mm^2^, 2.806 mm^2^, 0.791 mm^2^, and 0.353 mm^2^, respectively (Fig. 4). The anterior segment OCT taken at 1 day, 1 month, and 5 months after the surgery also showed a gradual decrease of the subcapsular cataract (Fig. 2). These results clearly indicate cataract reversal. Cataracts usually refer to the irreversible degeneration of lens material. Degeneration of structural proteins, which comprise the pathways of lens metabolism, plays a major role in cataract formation. In the case of primary nuclear cataracts, proteins may undergo nonenzymatic glycation, with an increase in nuclear molecules and with water soluble crystallins becoming water-insoluble. However, the development of subcapsular cataracts is mainly associated with permeability changes, such as damage to ion pumps and alterations in metabolic enzyme activities [13]. In subcapsular cataracts, external fluids enter through the lens capsule and develop limited fluid vacuoles, resulting in pumice-like opacities. In cases with severe ion pump damage, fluid may enter the lens nucleus and even form secondary nuclear cataracts. Subcapsular cataracts are known to be caused by a variety of factors, including aging, corticosteroids, iridocyclitis, and radiation. While these factors alter and impair ion pump function to a variable extent, we believe the instantaneous exposure of distilled water may induce transient permeability changes without irreversible damage, thus enabling the reversible cataract. As the lens cells repair and/or form, the vacuoles gradually disappear, and auto-regulation by active transport functions once again as normal membrane permeability is restored in newly formed lens cells. When the electrolytes normalize, lens clarity improves. However, if damage to the lens epithelium and fiber cells is so severe that the influx of water and disruption of cell membrane permeability persist, potassium ions, free amino acids, myoinositol, and glutathione are lost, and sodium and chloride ions accumulate. These electrolyte and biochemical imbalances lead to protein denaturation and cell death, eventually resulting in irreversible opacification [8, 14]. Although the cataract continuously decreased, the lens was not completely transparent and still showed a few opacities 5 months after the surgery. Considering the slowing rate of decrease since 1 month after surgery (Fig. 4), further follow-ups would be needed if partial irreversible opacification developed.

In conclusion, a cataract that developed after instantaneous intracameral exposure to distilled water was reversible. Because significant damage to the corneal endothelium can occur, surgeons should always pay special attention to the intraocular infusion fluid and check the original bottle. If distilled water is inadvertently infused, surgeons should promptly irrigate with a solution such as BSS. In addition, surgeons should perform sufficient follow-up before considering a surgical option such as keratoplasty or cataract surgery, keeping in mind that corneal edema and cataract caused by exposure to distilled water can be reversible.

## Abbreviations

BCDVA: best corrected distance visual acuity
BSS: Balanced salt solution
CCT: central corneal thickness
OCT: optical coherence tomography
SMILE: Small incision lenticule extraction
UDVA: uncorrected distance visual acuity
